# Water vapor desorption from silica gel in a combined drying and double-condenser compression refrigeration system

**DOI:** 10.1016/j.heliyon.2022.e09757

**Published:** 2022-06-21

**Authors:** Engkos Achmad Kosasih, Ahmad Zikri, Muhammad Irfan Dzaky

**Affiliations:** Department of Mechanical Engineering, Faculty of Engineering, Universitas Indonesia, Kampus UI Depok 16424, West Java, Indonesia

**Keywords:** Air dehumidification, Desorption, Activation energy, Desorption rate constant, Silica gel, Refrigeration

## Abstract

The drying method requires an effort to store food for a longer time. Some drying processes experience technical and economic weaknesses, mainly related to low efficiency, high energy costs, and decreased product quality. Various drying models have been studied to determine the suitability of heat and mass transfer analysis at drying rates in an air dehumidification scheme using different materials, one of which is silica gel. In this case, the researchers examined the effects of humidity, temperature, and airflow rate on the constant drying rate and activation energy of water desorption in silica gel using a packed bed dryer that was modified with a refrigeration system. This modified system aims to reduce specific energy consumption (SEC). The results demonstrate that the constant rate of water desorption in silica gel and the increase in air humidity cause a decrease in the constant value of the water desorption rate in silica gel. However, increases in the temperature and airflow cause an increase in the value of the constant drying rate for water desorption in silica gel, as they cause capillary evaporation. Meanwhile, the activation energy of water desorption in silica gel increases with decreasing air flow rate and increasing inlet air humidity. The attractive force acting on the water molecules from the surface force field on the surrounding walls becomes stronger if the air flow rate decreases or the air humidity increases. From the results and analysis, it is shown that the activation energy of water desorption in silica gel with significant air humidity and low flow rate, of 0.013 kg/kg d.a. (450 lpm), is the highest at 35.16 kJ/mol, whereas in silica gel with air humidity of 0.007 kg/kg d.a. (750 lpm), it is the lowest at 22.92 kJ/mol. Meanwhile, the dryer air flow rate, higher heater temperature, and lower air humidity improve the performance of the bed dryer against its evaporation rate and decrease the Specific Energy Consumption (SEC) value. SEC is also greatly influenced by the use condenser 1, which provides heater power savings of up to 79.1%. Thus, the system is expected to be applied to commercial drying systems that can work at low drying temperatures to maintain drying products and obtain low energy consumption.

## 1 Introduction

The COVID-19 pandemic has ravaged the world since the first cases were officially announced at the end of 2019. Subsequently, the number of confirmed positive cases increased and spread across all regions of the world. Several policies to tackle COVID-19 have been put into practice, including the implementation of large-scale social restrictions (PSBB). The implementation of PSBB has had an impact on almost all sectors of socioeconomic life. The food sector has been significantly affected by the COVID-19 pandemic. Efforts to ensure the availability of food must be studied. It is related to the goals of Sustainable Development Goals (SDG) number 2, namely, to end hunger, achieve food security, improve nutrition, and promote sustainable agriculture. One solution to this problem is to develop the food drying technology so that food becomes durable or long-lasting and microbiological or biochemical changes in the food can be inhibited (Yang, 2003). The drying process of a material generally requires a high temperature and high dry air flow rate to accelerate the drying process (Misha et al., 2012). Indonesia is a tropical area with a relatively high humidity level, and thus the development of drying technology is required. The drying process in tropical areas involves relatively high humidity levels. According to temperature measurements, tropical areas have an average temperature of 20 °C. In contrast, the average temperature in Indonesia can generally reach 35 °C with high humidity levels, which can reach 85% (hot and humid tropical climate) (Oh et al., 2017). Thus, the required drying process is quite varied, including the use of absorbent materials and different types of drying equipment.

In general, in Indonesians, the drying process is utilizing sunlight. In addition, with its natural potential, Indonesia certainly needs the development of drying technology. Indonesia's products that require drying include seafood, coffee beans, tea, grain, wheat, fruits, foodstuffs, and medicines. Meanwhile, there is palm oil waste in the energy and fuel sector in the form of palm oil shells that can be processed as fuel (75% of palm fruit is in the form of shells), in addition to low to medium quality coal, foodstuff, medicines, mineral seeds, and scent leaves (Suherman et al., 2020) (Mbegbu et al., 2021). Drying using sunlight is considered quite efficient because it does not cost money to carry out the drying process. However, drying with sunlight is highly dependent on the weather (El-Sebaii et al., 2002).

Before the last two decades, the development of drying technology occurred quite rapidly. However, the current situation barely shows any visible signs of progress. Technological progress is fueled by the energy crisis, consumer demand for better quality, and the start of significant research advances in fundamental and applied fields. Thousands of technical papers on the development of drying inventions have been published and are widely available. There is a synergistic effect between multidisciplinary disciplines, encouraging further advances in drying technology (Kudra and Mujumdar, 2009).

The drying method requires an effort to store food for a longer time. Some drying procedures have technical and economic flaws, which are mostly connected to inefficiency, high energy costs, and poor product quality. If the latent heat of vaporization of water is 2500 kJ/kg and the dryer efficiency is 50%, the total heat required for drying is 6875 MJ, a huge amount of energy. There are several techniques for dehumidifying drying air, including the use of adsorbents (such as synthetic silica, zeolite (3 A, 4A, and 13X), or the use of a refrigeration system (Djaeni et al., 2021)).

Research has been carried out to dry foodstuff in an energy-efficient manner by utilizing sunlight as a source of energy. The forced convection method was used to dry bananas that were thinly sliced and arranged in a tray. The drying air temperature varied between 35 and 60 °C. The bananas (10 kg) were dried with a humidity of 72% to 28% for four days, from 08.00 to 18.00. By utilizing a fan to evenly distribute the air to all parts of the dryer room, this method saves approximately 48% of drying time compared to drying with direct sunlight (Nabnean and Nimnuan, 2020). Drying of apples using solar air dryers has also been studied. The apples were sliced to 14 mm to facilitate the evaporation process. The tray used to place the apples was made of a perforated metal material so that the hot air could pass through the apple slices (Das and Akpinar, 2020).

Several of the studies conducted by experts in the field of drying are related to heat pump dryers or the combination of refrigeration systems with drying systems. A prototype drying system using a heat pump, the evaporator configuration used and comprises two levels, and R22 (chlorodifluoromethane) is used as the working fluid for the refrigeration system. The drying system consists of a closed loop, where the water vapor in the air resulting from the evaporation of the test material will condense in the evaporator. In this test, the material used for drying was water that was placed in a container. Water was used to simulate the wet material. As a result, up to 35% more heat can be absorbed by using a double evaporator when compared to a single evaporator (Chua and Chou, 2005).

The main advantages of using heat pump technology in the drying process are the potential for energy savings by recovering the evaporative and latent heat from the drying air and controlling the drying temperature and air humidity (Hall, 1988). The addition of a refrigeration system to the drying system can increase the energy consumption of the system. However, what needs to be considered is not the additional power required to operate the system but the specific energy consumption (SEC) value. When the SEC value obtained is low, the system can be profitable because the SEC of a drying system refers to the amount of energy required (Burheim et al., 2012) to evaporate water in the material (kg of water). Ebenezer has successfully tested the Integrated Refrigerator-Waste Heat Recovery Dryer (IRWHRD). IRWHRD is used to dry wet cotton fabrics with a temperature range of 49 °C and 2 °C. (Onyeocha et al., 2020).

Because the study by (Zikri et al., 2020) focuses solely on the drying system, the SEC refers to the amount of energy required to generate dry air. When the RSEC value for a refrigeration system (with R134a as the working fluid) is less than 1, the energy consumption for a dehumidifier at the same refrigeration system heating temperature is lower than for just heating. When the dry air that exits condenser 1 is preheated, the energy required to attain the desired temperature is reduced. This research also demonstrates that lowering the pressure in the evaporator while maintaining the same air flow rate results in a drop in water content due to evaporator condensation. The determined humidity ratio of 3.9 g/kg dry air at 100 lpm air flow rate and evaporator pressure of 2.6 bar demonstrates this. An SEC ratio of 0.928 is used to calculate this point, which is considered the best. Because of the low air flow rate, the entering air condenses more quickly in the evaporator refrigeration system. The air that emerges from the evaporator is becoming drier. By reducing the pressure in the evaporator (with lower Ratio Specific Energy Consumption (RSEC) assumptions), the reduced air flow rate creates air with a lower humidity ratio. Because the evaporator might freeze and prevent heat exchange, this situation will be challenging to attain.

Because of its huge pore surface area and high moisture adsorption capability, silica gel has been widely employed as a solid drying agent in dehumidification operations (Chang et al., 2004). Because the surface area and pore distribution of silica gel determine its adsorption ability, pore structure selection and management are critical. Many investigations have been undertaken on the influence of silica gel's pore structure on its water vapor adsorption capacity (Glaznev et al., 2010). Tashiro reported that the pore volume with a pore size of less than 2 nm could be related to the amount of adsorption at lower relative humidity (Tashiro et al., 2004). Chua found that the water vapor isotherm in silica gel with an average pore diameter of 2.2 nm could be explained by a Henry-type equation and the Toth isotherm model (Chua et al., 2002). Chung reported that the amount adsorbed per unit volume of silica gel increased with an increasing micropore zone (Chung and Chung, 1998).

Adsorption and desorption processes make up dehumidification technology using adsorption (Li et al., 2007a, Li et al., 2007b). In recent years, it has been proposed that the quantity of energy used in the regeneration process be used to offset the expenses of dehumidification/drying. As a result, the quantity of energy used during the regeneration process has become a significant issue. The size of the silica gel particle has no effect on the regeneration rate, according to Kuei-Sen (Chang et al., 2005). Chang claimed that modified silica gel could be regenerated in 0.5 hours at 90 degrees Celsius, and that silica gel with a greater pore capacity could be regenerated quickly (Chang et al., 2004). Ahmed demonstrated that when an inclined fluidized bed was applied, satisfactory regeneration rates were confirmed at a regeneration temperature of approximately 90 °C (Hamed, 2005). Singh studied the optimization of the regenerated air temperature and bed air velocity for minimum energy input (Singh and Singh, 1998). When the regeneration temperature was altered between 373 and 442 K for 4–16 hours, Rong-Luan discovered that if the water vapor adsorption isotherm in silica gel was type I, the absorption capacity at 298 K did not vary considerably (Yeh et al., 1992).

Multiple values of water desorption activation energy have been investigated in silica gel with varying pore sizes. (Li et al., 2007a, Li et al., 2007b). Furthermore, a two-dimensional simulation was performed using computational fluid dynamics (CFD) software for the drying process using silica gel (Jung et al., 2008). Mathematical modeling and simulations have been developed to estimate bed regeneration in silica gel (Manyumbua et al., 2014).

Based on the results of these studies, the authors attempted to analyze the heat and mass transfer phenomena more deeply at a drying rate in the air dehumidification scheme using the test material (silica gel) through an experimental method. In other words, the research focuses on the effects of humidity, temperature, and air flow rate on the constant drying rate and activation energy of water desorption in silica gel using a modified bed dryer with a refrigeration system. The heat source in this drying system is an electric heater and a condenser refrigeration system (heat recovery). The electric heater acts as the primary air heater, while the condenser heat source is integrated with the refrigeration system. This refrigeration system helps dry humid air at low temperatures in the evaporator refrigeration system. Furthermore, a condenser with a high temperature (output from the compressor) can be used as a heat source. Thus, the consumption of electrical energy can be minimized by utilizing a refrigeration system.

## 2 Material and methods

### 2.1 Sample preparation

**Figure 1:**
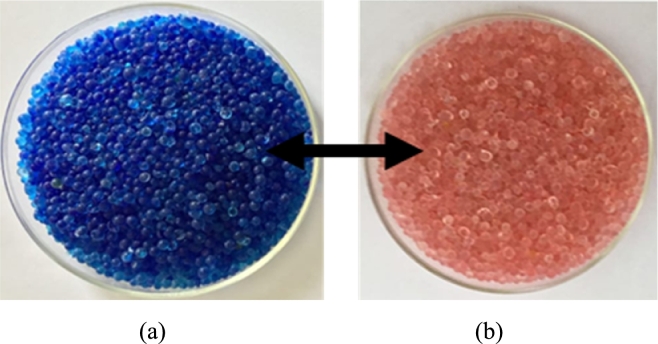
Sample preparation: (a) dry condition, (b) wet condition.

Preparation of the test material (silica gel) before the research was carried out to determine the initial mass of the dry silica gel at 100 g. Furthermore, after being moistened, the mass of silica gel reached 130 g (dry basis water content in silica gel was 30%). The prepared test material can be compared after the drying process is carried out using a modified bed dryer with a double-condenser compression refrigeration system. The silica gel used for this experiment was Fuji Davison type A. The thermophysical properties of this silica gel, as provided by Fuji Silysia Chemical Ltd., Japan, and from Chua et al. (Chua et al., 2002) (Li et al., 2007a, Li et al., 2007b), are listed in Table 1. The test sample (silica gel) is shown in Fig. 1.

**Table 1:** Thermophysical properties of silica gel.

Property	Type A
BET/N_2_ surface are (m^2^/g)	716±3.3
BET constant	293.8
BET volume STP (cm^3^/g)	164.5
Pore size (nm)	0.8∼5
Porous volume (cm^3^/g)	0.28
Micropore volume (%)	57
Mesopore volume (%)	43
Skeletal density (kg/m^3^)	2060
Particle bull density (kg/m^3^)	1306
Surface area (m^2^/g)	650
Average pore diameter (nm)	2.2
PH	5
Water content (mass %)	<2
Specific heat capacity (kJ/kg K)	0.921
Thermal conductivity (W/m K)	0.174

**Figure 2:**
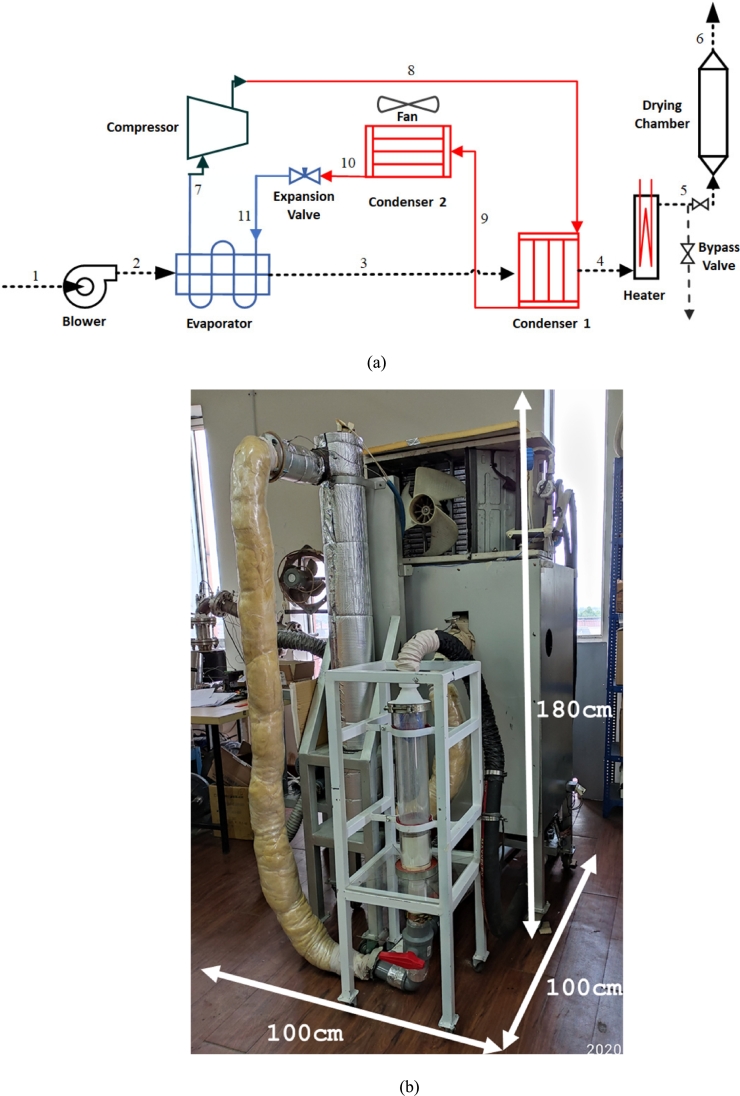
Experimental setup: (a) sketch, (b) final assembly.

Double condenser dryer was tested by varying the air flow rate, heating temperature, and humidity of the drying air. To get the optimal silica gel drying conditions, the air flow rate, heating temperature, and drying air humidity from the lowest, medium to the highest values. The data were collected using three different air flow rates (450, 600, and 750 lpm), four heater temperatures (60, 70, 80, and 90 °C), and four levels of air humidity entering the chamber (0.007, 0.0085, 0.010, and 0.013 kg/kg d.a.). The specific humidity variation (Y) is a function of temperature and relative humidity, Y=f (T, RH), based on the inlet temperature of the condenser through the refrigeration system, namely, 12.1, 15.1, and 18.1 °C, and the ambient temperature without the refrigeration system, 26.6 °C. The data collection scheme is illustrated in Fig. 2(a). However, the drying device consists of two systems related to the work procedure: a refrigeration system and a drying system. The two systems are described in the following subsections.

Based on Fig. 2, the main components of this drying system consist of a refrigeration system, a blower, and a drying chamber. In detail the capacity and components of the experimental setup can be seen Table 2 above.

**Table 2:** Components used in the experimental setup.

No	Component	Capacity/Dimension
1	Blower	1,3 m^3^/min
2	Air heater	3000 W
3	R134a Compressor	0,5 HP
4	Evaporator (Finned tube)	30 × 30 × 30 cm/(3/8 in tube)
5	Condenser 1 (Finned tube)	30 × 30 × 30 cm/(3/8 in tube
6	Condenser 2 (Finned tube)	55 × 48 × 1 cm/(1/4 in tube)
7	Needle valve	Max 6000 psi
8	Drying Chamber	Height = 50 cm, Outer Diameter = 11 cm, Inner Diameter = 10 cm

### 2.2 Refrigeration system

The refrigeration system used in this study consists of several main components, including a 0.5 HP compressor, condenser, expansion valve, and evaporator which are described in Table 2. The working principle of this refrigeration system is simply the same as the air conditioning system. However, today's method is given a condenser 1 and a blower. First, the compressor compresses refrigerant 134a so that there is an increase in pressure accompanied by an increase in temperature. Furthermore, the high-pressure and high-temperature refrigerant releases heat in condenser 1. Condenser 1 functions as heat recovery in the drying system.

Moreover, the refrigerant will go to condenser 2 to release further heat into the environment at a lower temperature. Condenser 2 is added to overcome the increased refrigeration system pressure caused by heat release in condenser 1. Refrigerant passing through the expansion valve will result in a drastic pressure drop and low temperature. As a result, the refrigerant will change phase from gas to liquid and is ready to absorb heat in the evaporator and will then be re-compressed by the compressor.

The refrigerant will absorb heat from the air exhaled by the blower in the evaporator. In the evaporator, there will be dehumidification of the air exhaled by the blower so that the exhaled air has a lower water content than before. Dry air will go through condenser 1 to absorb heat (preheating). If the target drying temperature has not reached the target, it will be heated with an electric heater. The drying air then goes to the drying chamber, which contains silica gel to be dried. In short, this system was equipped with two condensers connected in series in the refrigeration system.

In the first step, the blower sucks environmental air around the refrigeration system and sends it to the evaporator. Before entering the evaporator, the airflow rate was controlled through the blower rotation. The specific humidity of the environmental air (Y) is determined based on the dry bulb temperature and RH values in the environment. Furthermore, the calculation of the dry air mass flow rate (${\overset{˙}{m}}_{da}$) in the environment sucked by the blower, can use the following equation (1) and (2):

(1) $${\overset{˙}{m}}_{a}={\overset{˙}{m}}_{da}+{\overset{˙}{m}}_{v}$$

(2) $${\overset{˙}{m}}_{da}=\frac{{\overset{˙}{m}}_{a}}{1+Y}$$

The evaporator is a heat exchanger that decreases the temperature of the air that enters the environment and reduces air humidity. Humid air passes through the evaporator tube and transfers heat to the refrigerant, which has a temperature lower than the ambient air temperature. The evaporator absorbs heat ($Q_{\mathrm{Evap}}$) according to the following equation (3) or (4):

(3) $$Q_{\mathrm{Evap}}={\overset{˙}{m}}_{da}(h_{2}-h_{3})$$

(4) $$Q_{\mathrm{Evap}}={\overset{˙}{m}}_{\mathrm{ref}}(h_{7}-h_{11})$$

Furthermore, the dry and low-temperature air will come out of the evaporator and then flow to condenser 1 as an initial step of heating the air. The heat that rejected condenser 1 ($Q_{\mathrm{Cond},1}$) was completely absorbed by the air and can be obtained based on the following equation (5) or (6):

(5) $$Q_{\mathrm{Cond},1}={\overset{˙}{m}}_{da}(h_{4}-h_{3})$$

(6) $$Q_{\mathrm{Cond},1}={\overset{˙}{m}}_{\mathrm{ref}}(h_{8}-h_{9})$$

In the next stage, the dry air flows to the heater. There was a connection from condenser 1 to the heater in this air duct, and the excess heat was discharged into the surrounding air (environment) through condenser 2 by utilizing a fan. Furthermore, the hot and dry air from condenser 1 heated by the 3 kW electric heater. Furthermore, the dry and hot air flows into the drying chamber. The heat on the heater can be obtained using the following equation (7):

(7) $$Q_{\mathrm{Heater}}={\overset{˙}{m}}_{da}(h_{5}-h_{4})$$

The heater required massive heat for the material drying process, and the temperature was controlled in the chamber. However, the work of this heater was minimized by condenser 1. The air from this condenser has already attained a high temperature and dried before entering the heater chamber, which was a significant energy-saving step. To obtain the total energy consumption, it was necessary to measure the power of all the components based on the following equation (8):

(8) $$\mathrm{EC}=P_{\mathrm{Blower}}+P_{\mathrm{Fan}}+P_{\mathrm{Comp}}+Q_{\mathrm{Heater}}$$

To analyze the specific energy consumption of the dryer, the energy required to evaporate 1 kg of water must be measured. Therefore, the drying air enters the drying chamber has a specific air humidity value (Y), and the value of the evaporation rate can be calculated using the following equation (9):

(9) $${\overset{˙}{m}}_{\mathrm{ev}}=\frac{-dm}{dt}=\frac{-d(m_{dm}-m_{w})}{dt}$$

The value of $m_{dm}$ is constant for each test material, so the equation can also be written as equation (10)

(10) $${\overset{˙}{m}}_{\mathrm{ev}}=\frac{-dm_{w}}{dt}={\overset{˙}{m}}_{da}(Y_{6}-Y_{5})$$

Thus, the SEC value in the drying system can be calculated based on the following equation (11):

(11) $$\mathrm{SEC}=\frac{\mathrm{EC}}{m_{\mathrm{ev}}}$$

The performance of the drying system combined with the refrigeration system, that is, the ratio of specific energy consumption (RSEC), can be calculated based on the following equation (12):

(12) $$\mathrm{RSEC}=\frac{{\mathrm{SEC}}_{\mathrm{heater}+\mathrm{ref}}}{{\mathrm{SEC}}_{\mathrm{heater}}}$$

### 2.3 Drying system

This section presents the drying system was used. In short, the choice of a bed dryer was based on the drying criteria for dehumidification of air using the silica gel desiccant type, and thus the determination of the desiccant shape will not affect the drying system. The adoption of silica gel as a desiccant is because it can absorb moisture and liquid particles from a room with high temperature or water content without changing the condition of the substance and can be reused as needed (it is reusable).

**Figure 3:**
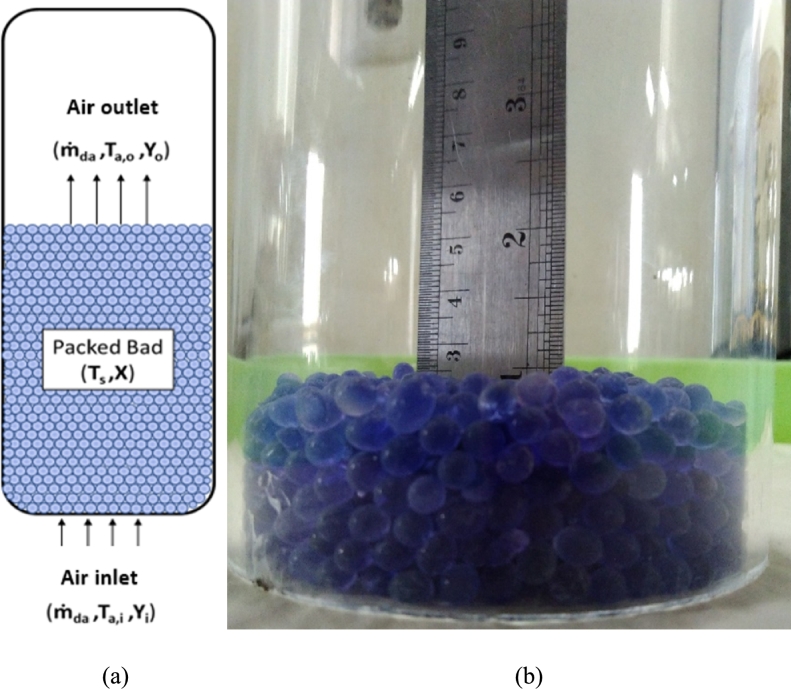
(a) Schematic of the drying chamber, (b) Height of silica gel in drying chamber.

After the refrigeration system process that previously ended in the heater, the air with a high and dry temperature flows into the drying room. Silica gel with 0% moisture content as much as 100 g was placed into a cylindrical drying chamber with a diameter of 100 mm to obtain a silica gel stack height of 25 mm. A schematic of the drying system in the drying chamber is shown in Fig. 3.

The variables in the air are the mass flow rate, temperature, and humidity ratio. In contrast, the variables in the packed bed are the desiccant temperature and water content in the desiccant. From these relevant variables, various equations can be formulated to analyze this modified drying system for mass balance in the system, including the mass balance of water vapor in the drying air and mass balance of water in the material. Therefore, it is necessary to know that the air flow rate at the inlet and outlet sides will carry a specific value of water vapor content (expressed as the humidity ratio) in the drying chamber.

Then, water vapor evaporates during the dehumidification process, and all the water vapor accumulates in the air, filling the drying chamber in the packed bed. Therefore, the rate of evaporation was obtained using the following equation (13) or (14):

(13) $${\overset{˙}{m}}_{\mathrm{ev}}=\frac{-dm}{dt}=\frac{-d(m_{dm}-m_{w})}{dt}$$

where $m_{dm}=\text{constant}$. Thus,

(14) $${\overset{˙}{m}}_{\mathrm{ev}}=\frac{-dm_{w}}{dt}={\overset{˙}{m}}_{da}(Y_{o}-Y_{i})$$

The humidity entering and leaving the drying chamber can be calculated using the function Y=f(T,RH) or by plotting the dry bulb temperature and relative humidity values on the psychometric chart.

Meanwhile, the evaporation rate can also be determined using the drying rate equation for the falling rate period. Thus, the drying rate constant of the bed dryer can be calculated for each variation, that is, the airflow rate, heater temperature, or humidity, using the following equation (15), (16) and (17):

(15) $$\frac{{\overset{˙}{m}}_{\mathrm{ev}}}{m_{dm}}=-\frac{dx}{dt}=k{\left(X-X_{e}\right)}^{\alpha }$$

where the desorption reaction is assumed to follow first-order kinetics, α=1. Then,

(16) $${\overset{˙}{m}}_{\mathrm{ev}}=m_{dm}k(X-X_{e})$$

or

(17) $${\overset{˙}{m}}_{\mathrm{ev}}=k(m-m_{e})$$

To find the value of the drying rate constant, the following equation can be used equation (18):

(18) $$\frac{dm_{\mathrm{ev}}}{\left(m-m_{e}\right)}=-kdt$$

If the two sides are integrated, then the equation becomes equation (19).

(19) $$\ln\frac{\left(m-m_{e}\right)}{\left(m_{o}-m_{e}\right)}=-kt$$

Simple linear regression, y=mx+c, is used, where y is the dependent variable or dependent variable, x is the independent variable or independent variable, the value of c is the intercept or intersection point, and m is the slope. We can use this step to determine the k value from the above equation. Where k is the slope of the equation, and t is the independent variable.

Linear regression is based on the adoption of the kinetic theory. In this case, the author decided to use a kinetic reaction order equal to 1 (α=1). Although the calculation can be made with exponential regression, unclear irregularities were generated when the authors performed the analysis using exponential regression. The proportion of variance in the dependent variable that can be predicted from the independent variable has a negative value, where for the value ($m-m_{e}$), it is not only exponential to the power kt ($e^{\left(k\;t\right)}$), but there are other variables. Therefore, in analyzing the drying rate constant of water vapor desorption in silica gel, it is more appropriate to use linear regression. The proportion of variance in the dependent variable that can be predicted from the independent variable has a proper value.

The activation energy is determined from the natural logarithmic curve k (ln k) vs. 1/T, where T is the drying temperature in Kelvin. The curve produces a straight-line equation as follows (Kosasih et al., 2020) using equation (20):

(20) y=mx+c

Based on the Arrhenius activation energy equation (Fiorentini et al., 2015), the relationship between ln k and 1/T is shown by the following equation (21) or (22):

(21) $$k=Ae^{\left(-\frac{\mathrm{Ea}}{R}\times \frac{1}{T}\right)}$$

or

(22) $$\ln k=(-\frac{\mathrm{Ea}}{R}\times \frac{1}{T})+\ln A$$

From equations (21) and (22), it can be obtained that y represents the value of ln k, and x represents the value of 1/T. The slope obtained is the value of −Ea/R., where the value of R is the ideal gas constant of 8.3145 J/mol.K. Thus the value of the activation energy can be calculated.

The energy consumption of the drying system is specifically carried out by measuring the total energy consumption of the evaporation of water from the test material. Based on the specifications of the measuring instrument used (Table 3), the uncertainty value of energy consumption is 2,49% based on the following equation (23):

(23) $$u_{\mathrm{EC}}^{2}={\left(\frac{\partial \mathrm{EC}}{\partial P_{\mathrm{Blower}}}\right)}^{2}u_{P_{\mathrm{Blower}}}^{2}+{\left(\frac{\partial \mathrm{EC}}{\partial P_{\mathrm{Fan}}}\right)}^{2}u_{P_{\mathrm{Fan}}}^{2}+{\left(\frac{\partial \mathrm{EC}}{\partial P_{\mathrm{Comp}}}\right)}^{2}u_{P_{\mathrm{Comp}}}^{2}{\left(\frac{\partial \mathrm{EC}}{\partial {\overset{˙}{m}}_{da}}\right)}^{2}u_{{\overset{˙}{m}}_{da}}^{2}+{\left(\frac{\partial \mathrm{EC}}{\partial T_{4}}\right)}^{2}u_{T}^{2}+{\left(\frac{\partial \mathrm{EC}}{\partial T_{3}}\right)}^{2}u_{T}^{2}$$

**Table 3:** Sensor measurement range and accuracy.

No	Sensor	Range	Accuracy
1	RH meter	0-100%	±3%RH for 20-80%RH; ±5%RH for 0-20%RH ∼ 80-100%RH
2	Thermometer	0-100 C	±1C
3	Air Flow meter	70-1400 lpm	±2%
4	Power meter	0-9999 kW	1 W

## 3 Result and discussion

### 3.1 Evaporation rate of silica gel

**Figure 4:**
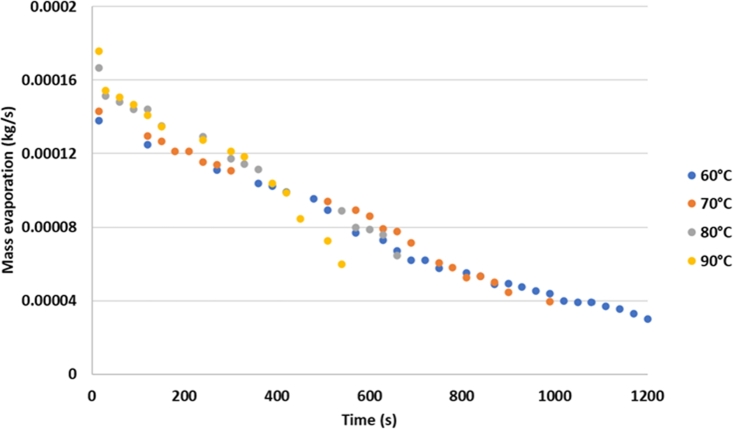
Graph of the effect to temperature and time on the evaporation of water vapor on silica gel.

There was a decrease in the water content in the tested silica gel due to the air flow rate, temperature, and air humidity entering the drying chamber. The graph of the evaporation rate versus time is shown in Fig. 4.

**Figure 5:**
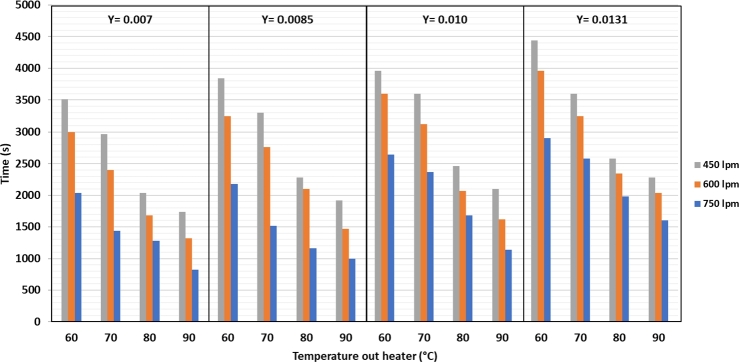
Drying time with variations in temperature, humidity of drying air, and air flow rate.

The graph shows the evaporation of water vapor on silica gel over time. The higher the drying temperature, the faster is the evaporation process of water in the silica gel. The higher temperature causes the water contained in the silica gel to evaporate more quickly. This means that the higher the drying air temperature is, the closer it is to the evaporation temperature of water at 1 atm pressure, which is 100 °C. The higher the drying air flow rate is, the faster the water mass transfer from the silica gel to the air. Based Fig. 5, the higher the air flow rate, the shorter the drying time. The humidity of the dryer air is an essential factor. The drier air causes the transfer of water from the silica gel to the air to be faster because there are differences in water concentration inside and on the silica gel surface against the drying air. The drying air binds water to the silica gel surface.

Fig. 5 shows that the higher the heater temperature is at the same air flow rate and humidity, the faster the drying time of silica gel. The drying time tends to be longer for the same heater temperature and air flow at different humidity levels. At the same heater temperature and drying air humidity, the higher the dryer air discharge is, the faster the drying time.

**Figure 6:**
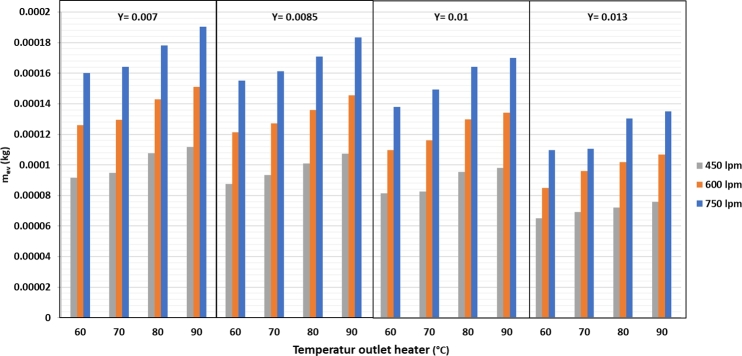
Mass of evaporation based on the temperature, humidity, and air flow rate variation.

From Fig. 6, it can be observed that the maximum rate of evaporation in silica gel is 0.00019 kg of water. It occurs at an air flow rate of 750 lpm, drying temperature of 90 °C, and air humidity of 0.007 kg/kg d.a. The lowest evaporation of silica gel is 0.00008 kg water at 450 lpm air flow rate, temperature of the drying air of 60 °C, and air humidity of 0.013 kg/kg d.a. The drying system uses a heater only, without dehumidification, and a heat recovery condenser.

### 3.2 Energy consumption of drying

**Figure 7:**
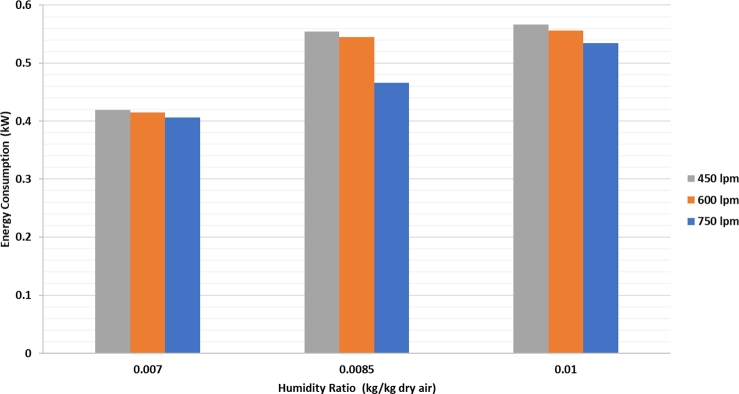
Energy consumption of the compressor vs. humidity of the dryer air at various dryer air flow rates.

Fig. 7 shows the energy consumption of the refrigeration system compressor, which increases with increasing humidity of the air at the same drying air flow rate. This is due to the larger opening of the expansion valve, which causes an increasing amount of expanded refrigerant to enter the evaporator. Further choking of the expansion valve causes less refrigerant to enter the evaporator, resulting in a drastic drop in pressure in the evaporator. This pressure drop causes a temperature drop in the evaporator tubes, which causes condensation in the humid air that passes through it.

**Figure 8:**
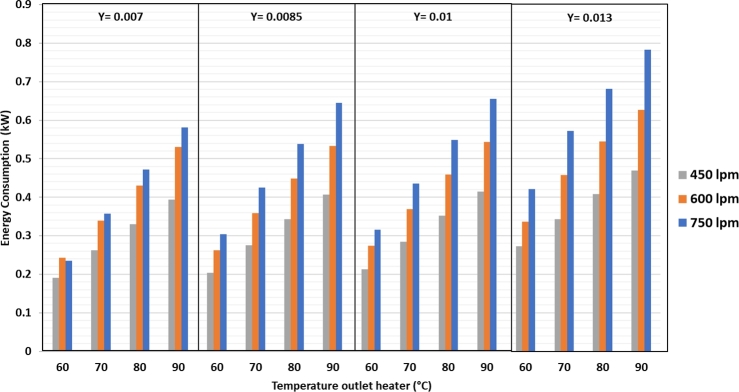
Heater energy consumption to drying air humidity at various dryer air flow rates.

At the same humidity in Fig. 8, a higher air flow rate through the evaporator causes the energy consumption of the average compressor to be lower. This is due to the discharge of drying air, which has a temperature higher than the wall temperature of the evaporator tube (the blower outlet temperature averages 30 °C). The high temperature difference and high air flow rate cause the heat received by the evaporator to be higher and increases the flow rate of the refrigerant, which changes its phase from liquid to vapor.

The trend of heater energy consumption in the drying equipment increased with an increase in heater temperature at the same humidity and air flow rate. In a drying system without refrigeration, the highest heating energy consumption is 783.1 W at an air flow of 750 lpm. The lowest energy consumption is 273 W at an air flow rate of 450 lpm.

When drying using a refrigeration system and heater, the heater energy consumption will be lower than that without using a refrigeration system. The highest heater energy consumption is 655 W under heater temperature conditions of 90 °C with a 750 lpm air flow rate. The lowest energy consumption of the heater is 191 W when the heater temperature is 60 °C and the air flow rate is 450 lpm. At the same heater temperature and air flow rate, the energy consumption of the heater exhibits an increasing trend. The exit temperature of condenser 1 increases when the humidity of the air decreases.

**Figure 9:**
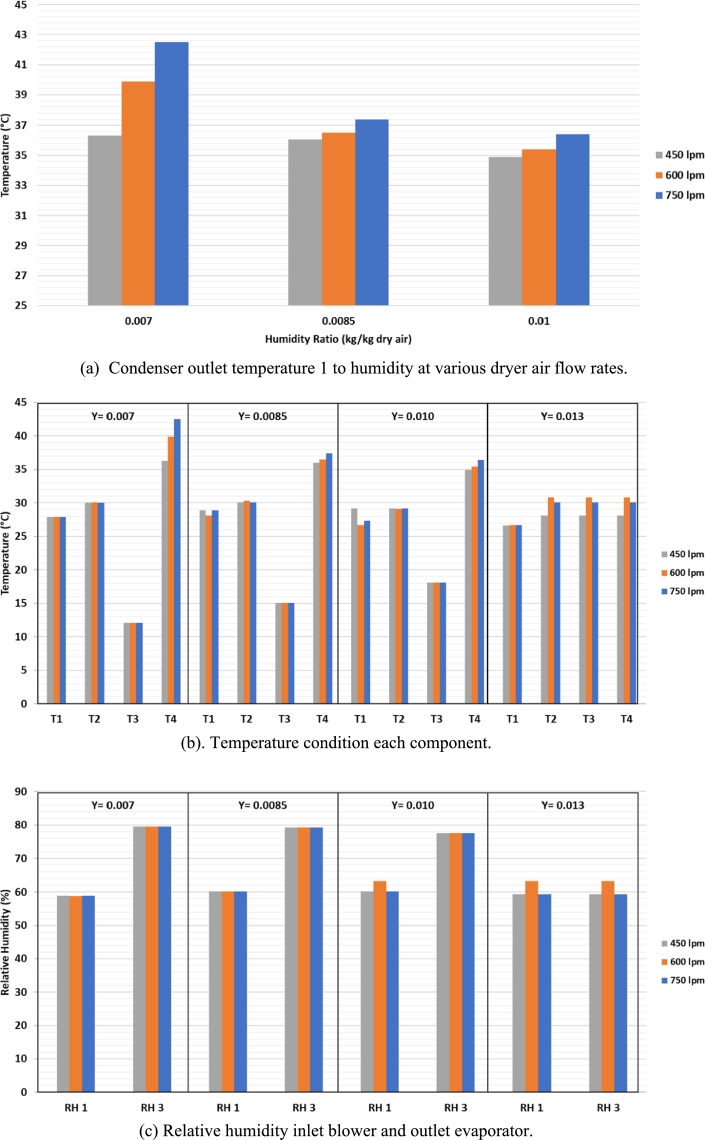
Graph of the (a) Condenser outlet temperature 1 to humidity at various dryer air flow rates, (b) Temperature condition each component, (c) Relative humidity inlet blower and outlet evaporator.

Fig. 9 (a) show that the outlet temperature of condenser 1 is inversely proportional to the humidity of the air produced by the evaporator during the dehumidification process. To achieve an air humidity of 0.007 kg/kg d.a., more throttling of the expansion valve is required at higher air flow rates. When the expansion valve is choked, it causes a pressure drop in the evaporator, which results in a decrease in temperature in the evaporator. This can cause the air that passes through the walls of the evaporator tube to condense and produce drier air out of the evaporator. The pressure in condenser 1 will increase when the expansion valve is throttled which causes a temperature rise in condenser 1. The increase in air flow rate also causes heat absorption and dehumidification of the air in the evaporator to increase and has an impact on increasing the pressure and temperature in the condenser 1. This is due to the rotation of the refrigeration compressor which tends to be constant so that it flows R134a relies on the throttling of the expansion valve which causes changes in temperature and pressure in the refrigeration cycle. Fig. 9 (b) shows the temperature conditions at the blower inlet (T1), blower outlet (T2), evaporator outlet (T3), and condenser 1 outlet (T4). The blower inlet temperature shows the average in each test condition at 27.7 °C, then a temperature increase at the blower outlet on average up to 29.7 °C. Then, at the evaporator outlet temperature, the humidity of the drying air is varied. So that the higher the humidity variation of the drying air, the higher the evaporator outlet temperature will be. Fig. 9 (c) RH in the blower inlet conditions obtained an average of about 60%. At the evaporator outlet, the RH value will decrease with increasing variations in air humidity value.

**Figure 10:**
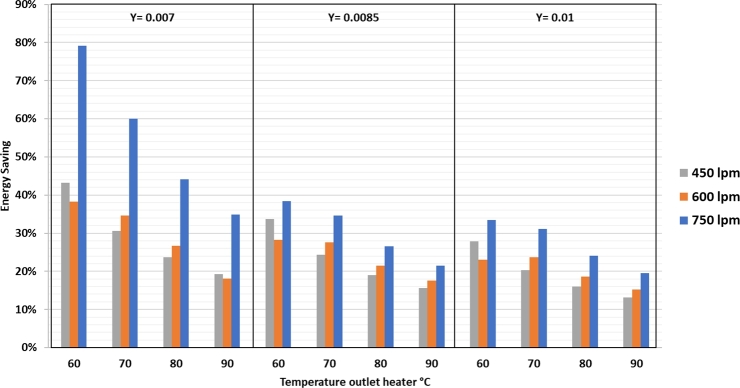
Energy recovery of condenser 1 against heater temperature and drying air humidity.

The percentage of heater power savings obtained by the addition of the refrigeration system by utilizing heat from condenser 1 can be observed in Fig. 10.

**Figure 11:**
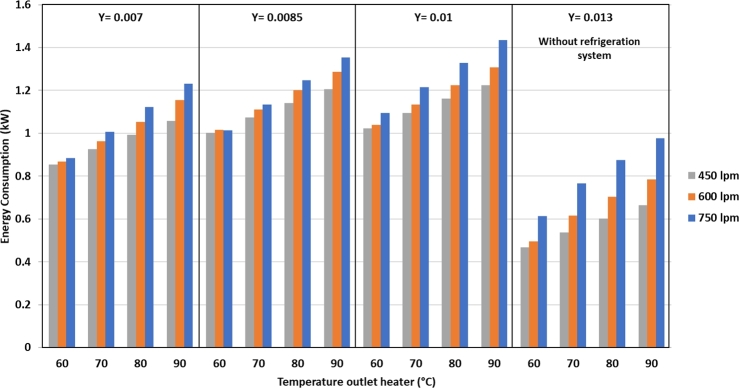
Total energy consumption against air flow rate, heater temperature and drying air humidity.

In Fig. 10, it is clear that there is a reduction in the heater power due to the addition of the refrigeration system. At 750 lpm air flow and 0.007 kg/kg d.a. humidity, the most significant energy saving at 79.1% is obtained at a heater temperature of 60 °C. At 450 lpm air flow and 0.01 kg/kg d.a. humidity, the power saving is 13.2%. Overall, the power required from the dryer to dry silica gel is shown in Fig. 11.

In Fig. 11 it is clear that the highest energy consumption using a refrigeration system is 1.43 kW, which occurs at 750 lpm air flow rate, 90 °C heater temperature, and air humidity of 0.01 kg/kg d.a. When the dryer uses the refrigeration system, additional power is required for the compressor and fan in condenser 2. The lowest energy consumption is 466 W with air flow rate of 450 lpm and heater temperature of 60 °C; in this case, the power is used only to operate the blower and heater, and the refrigeration system is not used. Based on the picture above, it can be seen that the energy consumption using a refrigeration system is much greater than without using a refrigeration system. However, by using a refrigeration system, the evaporation rate of water in silica gel will be greater than without using a refrigeration system at the same drying temperature. This condition is due to the drier air obtained after the dehumidification process, which draws more moisture from the silica gel than the air that is drained from the environment only by heating the heater. The difference in the concentration of water content in the drying air after going through the dehumidification process and the air in the environment, simply causes the water contained in the silica gel in saturated conditions to be carried away with the drying air faster because dry air can bind more water vapor.

**Figure 12:**
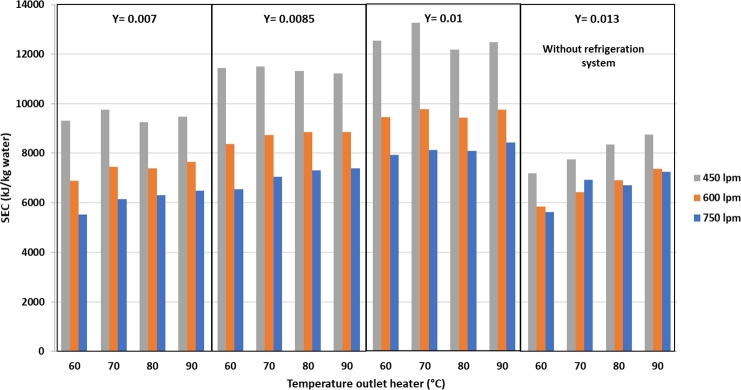
Specific energy consumption (SEC) against air flow rate, heater temperature, and drying air humidity.

**Figure 13:**
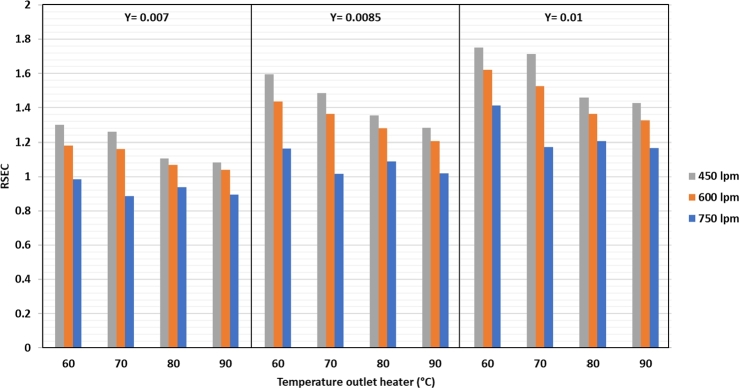
RSEC value against air flow rate, heater temperature, and drying air humidity.

The SEC value is the ratio between the total energy consumption and the mass of water vapor evaporated from the silica gel. The SEC was calculated when the water was at a maximum for instantaneous evaporation. Fig. 12 shows that the lowest SEC value is achieved using a refrigeration system of 5520 kJ/kg_water_ at an air discharge of 750 lpm, a heater temperature of 60 °C, and 0.007 kg/kg d.a. humidity. Therefore, it takes 5523 kJ of energy to evaporate 1 kg of water in the silica gel. The highest SEC value was 13268 kJ/kg water at an air flow rate of 450 lpm, heater temperature of 70 °C, and 0.01 kg/kg d.a. humidity. The tendency is that the higher the heater temperature and humidity at the same air flow rate, the higher is the SEC value. The higher the air flow rate at the same heater temperature and humidity, the lower is the SEC value. The RSEC value in Fig. 13 can be used to compare the dryer in combination with a refrigeration system with that without a refrigeration system.

From Fig. 13, it can be observed that the RSEC in the drying system combined with the refrigeration system has the lowest value of 0.88 under the air flow rate condition of 750 lpm, heater temperature of 70 °C, and humidity of 0.007 kg/kg d.a. The highest RSEC value is 1.75 with air flow rate of 450 lpm, heater temperature of 60 °C, and humidity of the dryer air of 0.01 kg/kg d.a. The graph trend in Fig. 13 shows that the higher the air flow rate and heater temperature at the same humidity, the lower the RSEC. Conversely, the higher the humidity at the same temperature and air flow, the higher is the RSEC value. The RSEC value below 1 means that the SEC of a drying system using the refrigeration system is smaller than that without the refrigeration system. This RSEC aims to see if the drying process at the same drying temperature and air flow rate can produce a better system using a refrigeration system for dehumidification of drying air. If the value is less than 1, then the silica gel drying process using a refrigeration system is considered profitable because the SEC value of a drying system with a refrigeration system is lower than without a refrigeration system.

### 3.3 Drying rate constant

**Figure 14:**
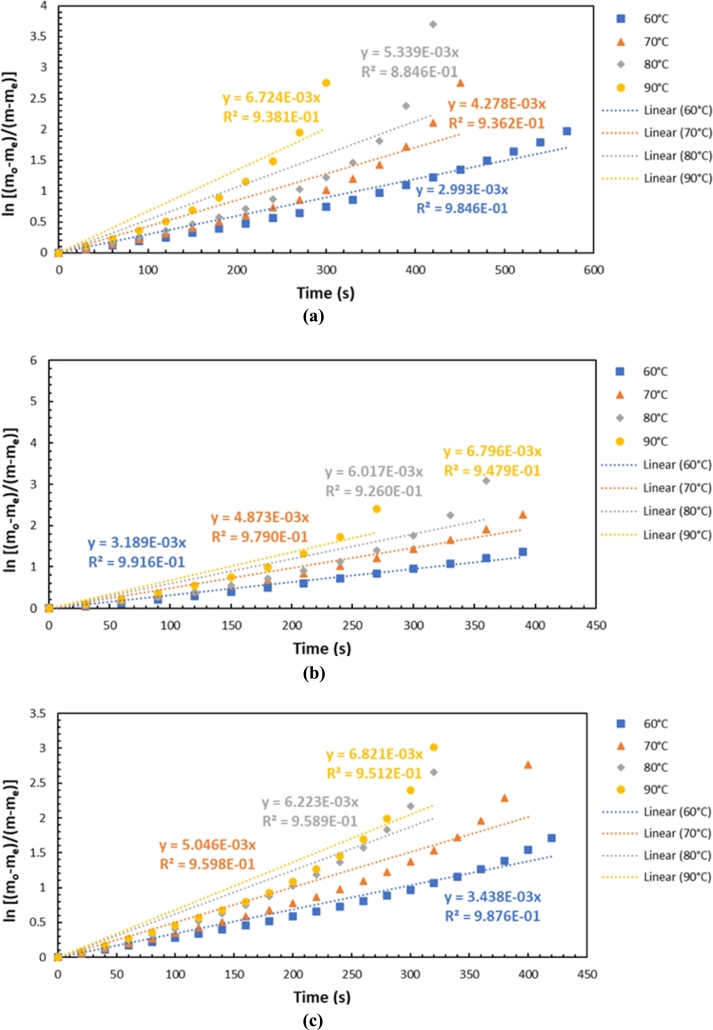
Linear dependence ln[(mo-me)/(m-me)] vs. time with air humidity of 0.007 kg/kg d.a. at air flow rate of (a) 450 lpm, (b) 600 lpm, (c) 750 lpm.

Fig. 14 shows the linear dependence between ln[(mo-me)/(m-me)] and time with an air humidity of 0.007 kg/kg d.a. at air flow rates of (a) 450 lpm, (b) 600 lpm, and (c) 750 lpm. For the other three humidity levels, 0.0085, 0.010, and 0.013 kg/kg d.a., the same trend is observed. Air humidity of 0.013 kg/kg d.a. is the condition of dryer air without a refrigeration system that flows into the drying chamber.

Further, we can observe that the graph plot forms a straight line with an average R square value of 90% of the total desorption at different airflow rates and humidity levels. The linear dependence model using the intercept function is suitable for describing the water desorption of the silica gel. Drying silica gel at a temperature of 60 °C is a condition that tends to be more stable (linear) because there is no damage to the material. So based on this graph can be approached by linearity regression.

Linear regression is based on the adoption of the kinetic theory. In this case, the author decided to use a kinetic reaction order equal to 1 (α=1). Although the calculation can be made with exponential regression, unclear irregularities were generated when the authors performed the analysis using exponential regression. The proportion of variance in the dependent variable that can be predicted from the independent variable has a negative value, where for the value ($m-m_{e}$), it is not only exponential to the power kt ($e^{\left(k\;t\right)}$), but there are other variables. Therefore, in analyzing the drying rate constant of water vapor desorption in silica gel, it is more appropriate to use linear regression. The proportion of variance in the dependent variable that can be predicted from the independent variable has a proper value.

**Figure 15:**
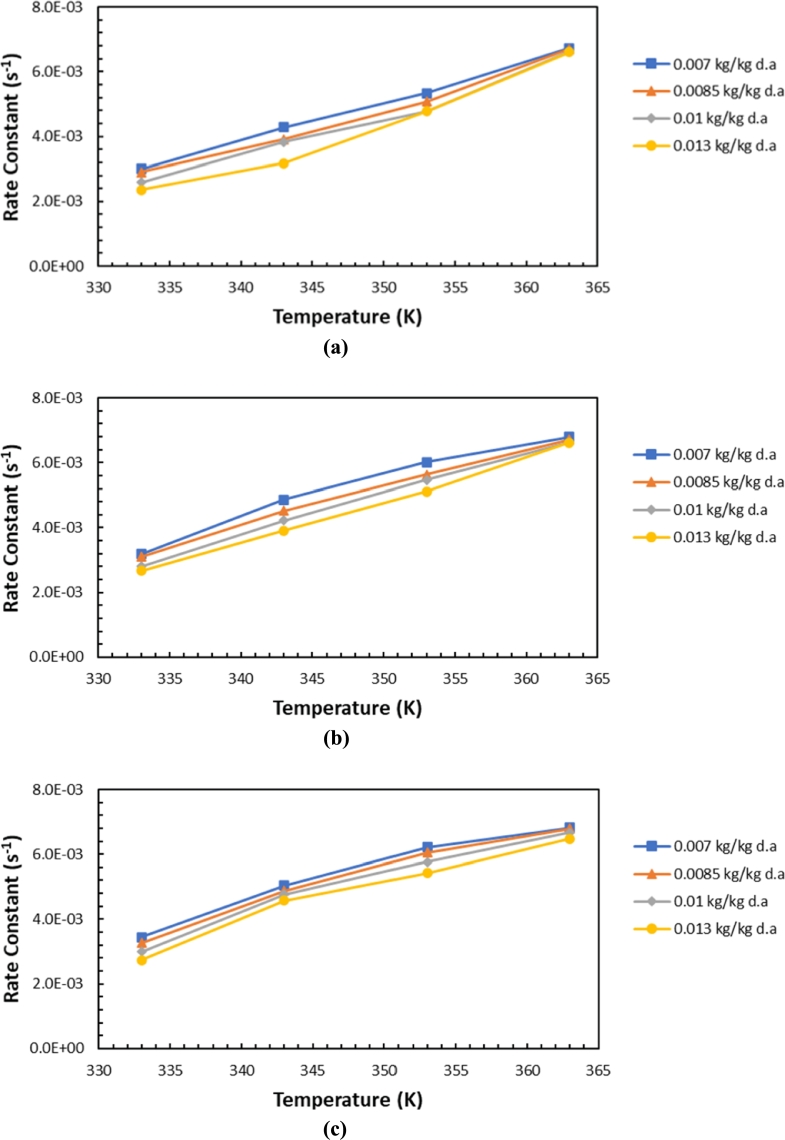
Variation of water desorption rate constant with different temperatures at air flow rates of (a) 450 lpm, (b) 600 lpm, (c) 750 lpm.

Fig. 15 shows the effect of temperature on the drying rate constant for water desorption at different inlet air humidity levels and varying air flow rates. The results show that silica gel's water desorption rate constants are very different for different temperatures, humidity levels, and air flow rates. However, at a temperature of 90 °C, the result does not demonstrate that the drying rate constants are quite different for each variation of air humidity or different air flow rates. The humidity of 0.007 kg/kg d.a. always obtains a sizable value in the water desorption rate constant as the temperature increases. It occurs at different flow rates, namely, 450, 600, or 750 lpm. In short, the increasing humidity of the air causes a decrease in the constant value of the water desorption rate in the silica gel. However, the impact of increasing temperature and air flow causes an increase in the drying rate constant for water desorption in silica gel. When the temperature reaches 90 °C and the maximum air flow rate is 750 lpm, there is a rapid increase in the water desorption rate constant in the silica gel owing to capillary evaporation at higher temperatures or large airflow rates.

For silica gel with inlet air humidity levels of 0.0085 and 0.010 kg/kg d.a., the variation of the desorption rate constant with temperature shows almost the same trend, especially at temperatures of 70 and 80 °C, which is related to the water desorption mechanism of this silica gel. Meanwhile, when the inlet air humidity is 0.013 kg/kg d.a., the variation in the silica gel water desorption rate constant with temperature shows a much different trend from the other three humidity levels. This is because the environmental humidity directly flows into the drying room without going through the refrigeration system.

In short, the inlet air humidity demonstrates desorption between the inner core and the next layer (multilayer) and capillary evaporation of the silica gel. As shown in Fig. 5, the curve of the variation of the drying rate constant in silica gel with temperature for water desorption can be divided into three different regions. The first region, where absolute humidity (Y) is less than 0.012 kg/kg d.a., shows that desorption is dominant in the inner core, and thus it shows a monotonic increase in the value of the desorption rate constant until it reaches a maximum. The second area is between 0.012 and 0.0216 kg/kg d.a., where the desorption rate constant does not change significantly. This may be due to the predominant multilayer desorption in the water desorption process in silica gel with such moisture.

Because the adsorbate–adsorbate interaction in the multilayer desorption process is usually weaker than the interaction between the adsorbate molecule and the desorbent surface, the water desorption process is slowed down to reduce the water content in the silica gel. The third region is when the absolute humidity (Y) is greater than 0.0216 kg/kg d.a., in which there is an increase in the rate constant, but it is not significant compared to that in the two regions described previously. Capillary evaporation is more difficult to occur, and hence the possibility of desorption only occurs in the outer layer (desorbent surface) of the silica gel.

### 3.4 Activation energy

**Figure 16:**
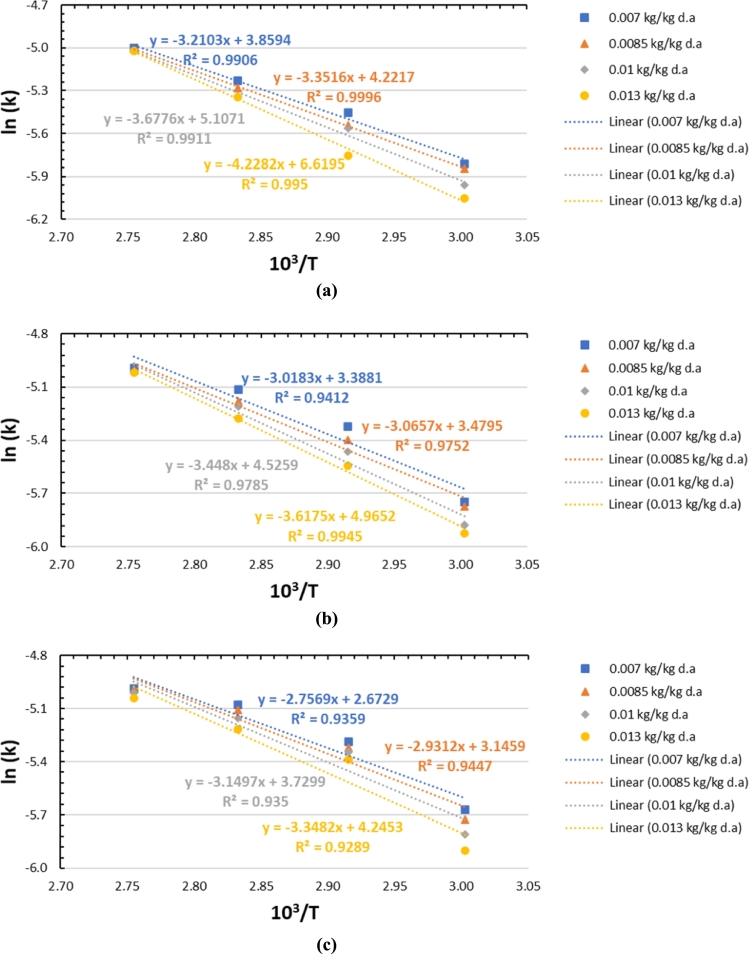
Linear dependence between ln⁡(k) and 10^3^/T at air flow rates of (a) 450 lpm, (b) 600 lpm, (c) 750 lpm.

The linear dependence between ln⁡(k) and 1/T at air flow rates of 450, 600, and 750 lpm with different air humidity levels is shown in Fig. 16. From the slope of these lines, the activation energy (Ea) can be observed; the value of A can also be obtained from the intersection of these lines on the y-axis, as shown in Fig. 16.

**Figure 17:**
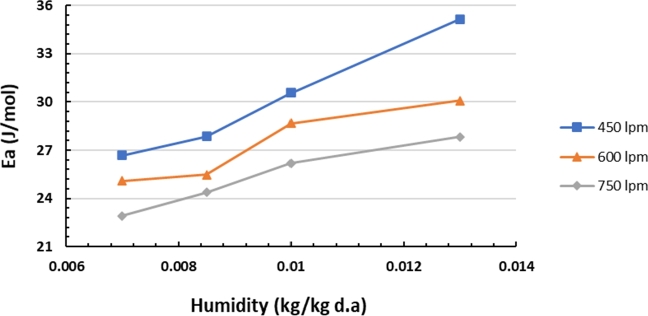
Activation energy vs. humidity at varying airflow rates.

The calculation of the water desorption activation energy in silica gel at air flow rates of 450, 600, and 750 lpm at different air humidity levels is presented in Table 4. The activation energy vs. humidity at each air flow rate is shown in Fig. 17. The higher activation energy values of water desorption in silica gel is indicated by occur with high humidity (0.013 kg/kg d.a.), which are 35.16 kJ/mol (at a flow rate of 450 lpm), 30.08 kJ/mol (at a flow rate of 600 lpm), and 27.84 kJ/mol (at a flow rate of 750 lpm).

**Table 4:** Activation energy with air flow rates of 450, 600, and 750 lpm.

Y	450 lpm	600 lpm	750 lpm
	Ea (kJ/mol)	Ea (kJ/mol)	Ea (kJ/mol)
0.007	26.692	25.096	22.922
0.0085	27.867	25.490	24.372
0.01	30.577	28.668	26.188
0.013	35.155	30.078	27.839

The trend graph in Fig. 17 shows that the desorption activation energy of water in silica gel increases with decreasing air flow rate. An increase in the activation energy of water desorption in silica gel occurs when the humidity of the drying air increases. The attractive force acting on the water molecules from the surface force field on the surrounding walls becomes stronger if the air flow rate is lower or the humidity is higher. As a result, the desorption activation energy of water in silica gel with high air humidity and/or low flow rate is 0.013 kg/kg d.a. (450 lpm), is the highest, while the humidity is 0.007 kg/kg d.a. (750 lpm), it is the lowest for the studied silica gel.

## 4 Conclusions

In this study, the silica gel drying process was observed in the drying chamber using a packed bed dryer. Thus, the characteristics of silica gel are obtained based on the drying conditions that have been tested. For the water desorption rate constant on silica gel, an increase in air humidity causes a decrease in the value of the water desorption rate constant on silica gel. However, the increase in temperature and air flow rate led to an increase in the drying rate constant for the desorption of water in silica gel. When the temperature reaches 90 °C and the maximum air flow rate is 750 lpm, there is a rapid increase in the water desorption rate constant in the silica gel due to capillary evaporation at higher temperatures or significant airflow rates. This study aims to obtain an energy efficient desiccant and characterization of silica gel, whose value can be used as a benchmark in the application of desorption of silica gel.

Meanwhile, the activation energy of desorption of water in silica gel increases with decreasing air flow rate and increasing intake air humidity. This is because of the attractive forces acting on the water molecules. The results and analysis showed that the desorption activation energy of water in silica gel with high humidity and the low flow rate was 0.013 kg/kg d.a. and 450 lpm, the highest value was obtained at 35.16 kJ/mol. For comparison, on silica gel with air humidity of 0.007 kg/kg d.a. and air flow rate of 750 lpm, has the lowest desorption activation energy value of silica gel water of 22.92 kJ/mol.

In general, the double-condenser refrigeration system assists the drying process and power savings of up to 79.1% are achieved at an air flow rate of 750 lpm and humidity of 0.007 kg/kg d.a. Under these conditions, the outlet temperature of condenser 1 is 42.5 °C. SEC tends to increase with increasing heating temperature and drying air humidity at the same airflow rate. Using a refrigeration system, the lowest RSEC value is 0.88 (drying air flow rate 750 lpm, heating temperature 70 °C, and drying air humidity 0.007 kg/kg d.a.). Under conditions of a drying air flow rate of 750 lpm, the humidity of 0.007 kg/kg, and various drying temperatures, RSEC values below 1 were obtained. This drying system with a double condenser refrigeration system can be relied on to get a drying process at low temperatures and low energy consumption.

## Declarations

### Author contribution statement

**Engkos Achmad Kosasih:** Conceived and designed the experiments; Analyzed and interpreted the data.

**Ahmad Zikri, Muhammad Irfan Dzaky:** Conceived and designed the experiments; Performed the experiments; Analyzed and interpreted the data; Contributed reagents, materials, analysis tools or data; Wrote the paper.

### Funding statement

This work was supported by 10.13039/501100006378Universitas Indonesia through Hibah Publikasi Terindeks Internasional (PUTI) Q1 2020 (NKB-1429/UN2.RST/HKP.05.00/2020).

### Data availability statement

Data will be made available on request.

### Declaration of interests statement

The authors declare no conflict of interest.

### Additional information

No additional information is available for this paper.
